# Greetings from the first Editor‐in‐Chief

**DOI:** 10.1002/deo2.1

**Published:** 2021-01-18

**Authors:** Takao Itoi


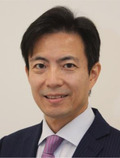


I am the Chair and Professor of Gastroenterology and Hepatology, Tokyo Medical University. It is my great honor to be designated as the first Editor‐in‐Chief (EIC) of DEN Open, the official journal of Japan Gastroenterological Endoscopy Society (JGES) and sister journal of Digestive Endoscopy (DEN), which has 4.774 Impact Factor (IF) value in 2019. I would like to express my sincere gratitude to the arrangement committee members of DEN Open, Prof. Haruhiro Inoue, the President of JGES, Prof. Shinji Tanaka, the Vice‐president of JGES, and Prof. Takayuki Matsumoto, the current EIC of DEN, for their tremendous effort to create the concept of DEN Open.

I contributed to DEN as an associate editor between 2012 and 2015 with the former EIC, Prof. Choitsu Sakamoto, and Prof. Hisao Tajiri, the former President of JGES. In 2012, they created the “young” editorial team for new DEN in order to make DEN a leading endoscopy journal in Asia‐Pacific region. Since then, young editors have been willing to edit to the best of their abilities. As a result, DEN has become widely accepted as an internationally dedicated journal of gastrointestinal (GI) endoscopy. Nowadays, DEN is also getting popular as one of the friendly GI endoscopy journals in the world, like “Endoscopy” and “Gastrointestinal Endoscopy”.

Recently, the number of submissions to endoscopy journals is increasing year by year. DEN has been accepting a considerable number of manuscripts, including Review article, Original article, Video article, etc. However, the number of publications is limited due to journal space. Thus, in this moment, we will open the new journal, DEN Open, in order to further distribute the outstanding and sophisticated manuscripts in GI endoscopy for researchers and readers in the world, particularly in the Asia‐Pacific region. We believe that the new space of DEN Open will be able to contribute not only to endoscopists, physicians, and surgeons, but also to the industry‐academia collaborators in endoscopy. In order to be accepted in the world, we will aim for early registration as an accepted journal in PMC (former “Pub‐Med Central”).

Despite publishing more, DEN Open will uphold the quality of the journal. Nowadays, responsible conduct of research, including research ethics and integrity, is required for clinical and epidemiological researches. Such conduct is also required for articles published in medical journals. While CONSORT requirement is well known as the one necessary for prospective clinical trials, other statements, including STROBE (STrengthening the Reporting of OBservational Studies in Epidemiology) and RECORD (REporting of studies Conducted using Observational Routinely‐collected Data), may become mandatory for articles published in DEN Open. In addition to shortening in time and appropriate assessment for peer‐review, DEN Open will introduce those statements in the instruction for authors.

The memorable first editorial board members of DEN Open are promising endoscopists and gastroenterologists who will make DEN Open sophisticated and popular like DEN. Furthermore, senior advisory board members from DEN will also help us as strong supporters and guide us in the right direction. In addition, I deeply appreciate the secretariat team of JGES in managing the new journal. I am very happy to experience the best start of a new journal as an EIC of DEN Open.

Finally, I would like to ask all endoscopists, gastroenterologists and surgeons in the world for further active participation in DEN Open. So, let's get started!

1 September, 2020

